# Angle-closure Glaucoma in a Myopic Patient Precipitated by Sexual Excitation: A Case Report

**DOI:** 10.5005/jp-journals-10028-1259

**Published:** 2018

**Authors:** Marko Popovic, Matthew B Schlenker

**Affiliations:** 1 Undergraduate Medical Education Program, Faculty of Medicine, University of Toronto, Toronto, Ontario, Canada; 2 Department of Ophthalmology and Vision Sciences, University of Toronto, Toronto, Ontario, Canada

**Keywords:** Acute angle-closure glaucoma, Endocyclophotoplasty, Goniosynechialysis, Myopia, Optical coherence tomography

## Abstract

**Aim:**

Herein, we report a case of a 55-year-old male who presented with intermittent acute-on-chronic angle-closure glaucoma triggered by sexual excitation.

**Background:**

Sexual excitation is an uncommon cause of pupillary block and angle closure attack.

**Case description:**

A 55-year-old male with a history of myopic laser in situ keratomileusis (LASIK) presented with a volatile intraocular pressure (IOP) and blurred vision over the last seven years. He was particularly symptomatic following sexual excitation. Examination revealed an IOP of 36 mm Hg and best-corrected vision of 20/80 OD, with bilateral closed angles and a double hump sign on gonioscopy. There were advanced glaucomatous changes OD and mild-to-moderate changes OS on optical coherence tomography. Following an exploration of potential options, it was chosen to proceed with OD lens-based surgery, goniosynechialysis and endocyclophotoplasty. During OD recovery, the patient reported an episode of visual blurring OS secondary to sexual excitation, which was consistent with pupillary block and angle closure attack on examination. Initially managed with acetazolamide and laser peripheral iridotomy, he eventually underwent the same surgical procedure OS as for OD. Over 1-year of follow-up, he has achieved a stable IOP and excellent visual acuity bilaterally.

**Conclusion and clinical significance:**

This case highlights the importance of a thorough history, with the understanding that sexual excitation can precipitate angle-closure glaucoma. Gonioscopy must be performed even in the setting of myopia and a deep anterior chamber, and the double hump sign must be assessed. Appropriate education surrounding the risks of sexual activity in angle closure suspects is advised.

**How to cite this article:**

Popovic M, Schlenker MB. Angle-closure Glaucoma in a Myopic Patient Precipitated by Sexual Excitation: A Case Report. J Curr Glaucoma Pract 2018;12(3):142-144.

## BACKGROUND

Sympathetic stimulation during sexual activity may lead to pupillary dilation.^1,2^ In eyes with occludable angles, this dilation may trigger pupillary block and an angle closure attack.^2^ We report a case of intermittent acute-on-chronic angle-closure glaucoma triggered by sexual excitation.

## CASE REPORT

**Figs 1A and B:**
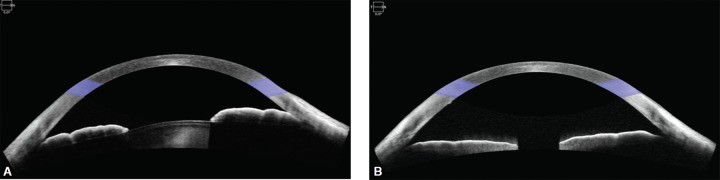
(A) December 2016, OD–Closed angles visualized on preoperative anterior segment optical coherence tomography; (B) February 2017, OD–Open angles visualized on postoperative week 1 anterior segment optical coherence tomography

**Figs 2A and B:**
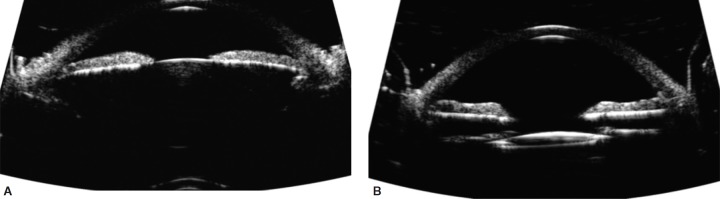
(A) December 2016, OD–Closed angles with plateau iris visualized on preoperative ultrasound biomicroscopy; (B) September 2017, OD–Open angles visualized on postoperative ultrasound biomicroscopy

In December 2016, a 55-year-old male was referred for volatile IOP with a history of myopic LASIK bilaterally and photorefractive keratectomy (PRK) enhancement OS. Seven years ago, the patient first noted episodes of blurred vision and halos lasting up to 2 hours, OD > OS, following sexual excitation. Best corrected visual acuity (BCVA) was 20/80 OD and 20/25 OS. The pupillary response was sluggish OD > OS. IOP was 36 mm Hg OD and 24 mm Hg OS on no topical medications. Anterior segment exam showed 2 + stromal corneal edema OD and 1 + OS. The anterior chamber was deep centrally and shallow peripherally. There was trace anterior segment reaction and trace nuclear sclerosis. Angles appeared closed bilaterally on gonioscopy. Dynamic gonioscopy showed scattered peripheral anterior synechiae OD > OS and a double hump sign. There was trace pigment in the trabecular meshwork. Optic nerve, optical coherence tomography (OCT) and visual field assessment demonstrated advanced glaucomatous changes OD and mild-to-moderate changes OS. Anterior segment OCT showed a deep anterior chamber with minimal lens vault, iris trabecular meshwork contact, and minimal pupillary block (Fig. 1). Ultrasound biomicroscopy demonstrated closed angles and anteriorly positioned prominent ciliary processes (Fig. 2).

Various treatment modalities were explained, including pilocarpine, laser peripheral iridotomy (LPI), laser iridoplasty, lens-based surgery, goniosynechialysis, endocyclophotoplasty, trabecular bypass procedures, and sub-conjunctival filtering procedures. We proceeded with lens-based surgery with goniosynechialysis and endocyclophotoplasty, pilocarpine 2% and travoprost-timolol 0.004%/0.5% were started. OD surgery went uneventfully with the mild cataract removed and a ZCBOO IOL implanted targeting distance. Two hundred degree of endocyclophotoplasty was performed nasally through the temporal incision with a curved 19-gauge probe.^3^ Total 270° of goniosynechialysis was performed using an Ocular Ahmed 1.5X Surgical Gonio and 25-gauge MST iris microforceps. Postoperatively, he was left on travoprost-timolol 0.004%/0.5%, pilocarpine 2% for the first 2 months, moxifloxacin 0.5% tid for the first week, nepafanac 0.1% tid for the first month, and prednisolone 1% q2h for the first week with a gradual taper over 6 weeks. He has achieved 20/20 UCVA, and his IOP has ranged from 7 to 12 mm Hg on travoprost-timolol 0.004%/0.5%. His postoperative anterior segment OCT and UBM show open angles and a well-centered 1-piece IOL in the capsular bag (Figs 1 and 2).

During OD recovery, the patient reported an episode of visual blurring OS secondary to sexual excitation. On examination, the IOP was 7 mm Hg OD and 40 mm Hg OS, with a fixed, mid-dilated pupil and closed angles with pupil block OS. The patient was treated with acetazolamide 500mg and an LPI. This reduced the IOP to 7 mm Hg OS and broke the acute angle-closure attack. The patient underwent lens-based surgery with goniosynechialysis and endocyclophotoplasty OS. He achieved J1 reading vision (near target for monovision) and a stable IOP after one-year of follow-up. He has developed scattered peripheral anterior synechiae over time, and we may consider iridoplasty or slit-lamp goniosynechialysis if there is future progression.

## DISCUSSION

In 2007, Ramasamy and colleagues reported a case of acute angle-closure glaucoma precipitated by sexual intercourse and administration of sildenafil citrate.^1^ A 71-year-old male presented with a painful loss of vision after taking sildenafil hours earlier. Visual acuity was hand motion with an IOP of 60 mm Hg and closed angles on gonioscopy. Following LPI and phacoemulsification with viscocanalostomy, his symptoms and vision improved. He achieved a BCVA of 20/20 and an IOP of 12 mm Hg.

Friedberg and Fox described three women aged 37, 45 and 55 years who developed blurred vision resulting from angle-closure glaucoma induced by sexual stimulation.^4^ Each patient described transient blurred vision lasting minutes to hours during sexual arousal. All patients underwent initial bilateral LPI, with one needing bilateral laser iridoplasty, the second requiring trabeculectomy bilaterally and the third receiving trabeculectomy OD and laser iridoplasty OS afterward.

Ritch et al. reported on a 34-year-old Caucasian woman with a past family history of angle-closure that presented with pain, blurred vision and halos for 2 days.^5^ These recurrent episodes were associated with orgasm and resolved spontaneously. On examination, she was hyperopicwith a best-corrected visual acuity of 20/25 OU and an IOP of 12 mm Hg OD and 17 mm Hg OS. Gonioscopy demonstrated appositional angle-closure OD, which responded to LPI and peripheral iridoplasty.

## CONCLUSION AND CLINICAL SIGNIFICANCE

This case highlights the importance of a thorough history, with the understanding that sexual excitation can precipitate angle-closure glaucoma. Gonioscopy must be performed even in the setting of myopia and a deep anterior chamber, and the double hump sign must be assessed. Appropriate education surrounding the risks of sexual activity in angle closure suspects is advised.
